# Remembering stress, shaping resilience: plant adaptation across scales

**DOI:** 10.1042/EBC20260069

**Published:** 2026-07-30

**Authors:** Estrella Luna

**Affiliations:** School of Biosciences, University of Birmingham, B15 2TT Birmingham, U.K.

**Keywords:** Climate change, Environmental stress, Forest resilience, Microbiome, Plant immunity, Stress memory

## Abstract

Plants face unprecedented environmental challenges arising from climate change, including increasing temperatures, altered nutrient availability, shifting seasonal patterns, and intensified biotic pressures. The present editorial introduces the special issue *Plant Adaptation to Changing Environments*, which brings together reviews examining plant adaptation from molecular to ecosystem levels. Collectively, these articles highlight how resilience emerges through the integration of signalling networks, metabolic regulation, developmental plasticity, ecological interactions, and environmental history. Together, they emphasise the need for interdisciplinary approaches to understand, predict, and enhance plant resilience in a rapidly changing world.

The accelerating pace of global environmental change presents one of the greatest challenges facing plants and the ecosystems they support. Rising temperatures, altered precipitation patterns, increasing atmospheric carbon dioxide concentrations, changing nutrient cycles, and the emergence of novel pests and pathogens are reshaping the conditions under which plants have evolved. As sessile organisms, plants cannot escape these pressures. Instead, they rely on remarkable physiological, biochemical, molecular, and ecological mechanisms that enable them to perceive environmental change, adjust their responses, and maintain fitness under fluctuating conditions. Understanding these adaptive processes has never been more urgent, both for safeguarding natural ecosystems and for ensuring future agricultural productivity and food security.

This special issue of *Essays in Biochemistry*, entitled *Plant Adaptation to Changing Environments*, brings together reviews that collectively examine how plants respond to environmental challenges across biological scales, from molecular signalling and epigenetic regulation to ecosystem interactions and forest resilience. While the topics span diverse disciplines, a common theme emerges throughout the collection: plant adaptation is a dynamic, integrative process that relies on the interaction of multiple biological systems operating across space and time.

Several contributions focus on the concept of defence priming and memory, mechanisms that allow plants to respond more effectively to future stresses following prior exposure. In 'Plant Defence Priming and Memory Mechanisms under Climate Change' [1], the authors synthesise current understanding of how priming operates under increasingly complex environmental conditions, highlighting the interactions between signalling pathways, epigenetic regulation, physiological trade-offs, and climate-related stressors. Complementing this broader perspective, 'From Early Defence Priming to Lasting Memory: Developmental and Seasonal Dynamics in Trees' [2] explores how priming responses vary throughout the lifespan of long-lived woody species. This review emphasises that defence responsiveness is not static but is profoundly influenced by developmental stage, seasonal cycles, and environmental history, providing important insights into how perennial plants may adapt to changing environments over decades or even centuries.

At the centre of many adaptive responses lies redox regulation. Reactive oxygen species (ROS), once viewed primarily as damaging metabolic by-products, are now recognised as fundamental signalling molecules that coordinate plant growth, development, and stress responses. The review 'Reactive Oxygen Species and Oxidative Signalling in Plants' [3] provides a comprehensive overview of ROS biology, highlighting their role in local and systemic signalling, post-translational regulation, and the emerging importance of ROS-driven liquid–liquid phase separation in controlling cellular responses. The mechanisms discussed in this review underpin many of the adaptive processes described throughout the Special Issue, demonstrating the central role of redox signalling in environmental responsiveness.

The translation of fundamental knowledge into practical applications is addressed in 'Developing Physical Seed Priming as a Practical Tool for Stress-Resilient Crop Production' [4]. Seed priming represents one of the most promising strategies for enhancing crop establishment and stress tolerance. The authors explore how physical treatments, including radiation and non-thermal plasma technologies, may induce beneficial physiological changes without some of the limitations associated with conventional hydration-based priming methods. As agriculture faces increasing climatic uncertainty, such approaches offer potential pathways towards sustainable enhancement of crop resilience.

Adaptation to environmental change also depends on the efficient acquisition and management of essential resources. Nitrogen availability remains one of the most important factors limiting plant productivity worldwide. In 'Plant Metabolic Adaptation to Nitrogen Scarcity: The Role of Biological Nitrification Inhibitors' [5], the authors examine how plants actively shape nitrogen cycling within the rhizosphere through the release of specialised metabolites that suppress microbial nitrification. This review highlights the sophisticated biochemical strategies plants employ to influence their surrounding environment and identifies exciting opportunities for developing more sustainable agricultural systems through improved nitrogen use efficiency.

A recurring theme across the collection is the recognition that plants do not function as isolated organisms. Instead, they exist within complex ecological networks that influence their performance and adaptive capacity. This concept is explored in 'The Plant Holobiont: Integrating Molecular Priming and Ecological Legacies for Climate-Adaptive Immunity' [6]. The authors argue for a broader view of plant immunity that incorporates interactions with beneficial microbes, soil communities, and environmental conditions. By linking molecular mechanisms with ecological processes, the review provides a valuable framework for understanding resilience in increasingly unpredictable environments.

Climate change poses particular challenges for forest ecosystems, where long generation times can limit the speed of adaptation. Two reviews address this issue from complementary perspectives. 'Forest Tree–Pathogen Interactions under Climate Change' [7] examines how drought, heat, emerging pathogens, and other stressors interact to shape tree health and survival. The review highlights the importance of integrating genetics, multiomics approaches, ecology, and climate modelling to predict future forest resilience. Meanwhile, 'Winter Climate Change in the Boreal Forest: What Does It Mean for Forest Tree Seedlings?' [8] focuses on the often-overlooked role of winter processes in forest regeneration. By examining how changing snow regimes, frost dynamics, and winter carbon cycling affect seedling establishment, this review reminds us that climate impacts extend beyond the growing season and that successful adaptation requires a year-round perspective.

Taken together, the contributions in this Special Issue illustrate the remarkable complexity of plant adaptation to changing environments. They demonstrate that resilience emerges not from any single mechanism, but from the integration of signalling networks, metabolic adjustments, developmental plasticity, ecological interactions, and evolutionary processes. At the same time, they highlight important knowledge gaps that must be addressed if we are to predict and manage plant responses under future environmental scenarios. I hope that this collection stimulates further interdisciplinary research and fosters collaboration across molecular biology, biochemistry, ecology, forestry, and agricultural sciences. Such integration will be essential for developing innovative strategies to enhance plant resilience and ensure sustainable food and forest systems in a rapidly changing world.

## Open Access

Open access for this article was enabled by the participation of the University of Birmingham in an all-inclusive Read & Publish agreement with Portland Press and the Biochemical Society under an agreement with JISC.

## Abbreviations

ROS: reactive oxygen species
